# Anatomical Variation in the Formation of the Median Nerve With Triple and Quadruple Roots of Origin: A Cadaveric Case Report

**DOI:** 10.7759/cureus.94612

**Published:** 2025-10-15

**Authors:** Yash Yadav, Rahul Ray, Azmi Mohsin, Shashwat Arora, Waqas Alauddin, Brishabh R Prajesh, Ishita Singh

**Affiliations:** 1 Anatomy, Naraina Medical College and Research Centre, Kanpur, IND; 2 Medicine, Naraina Medical College and Research Centre, Kanpur, IND; 3 Physiology, Naraina Medical College and Research Centre, Kanpur, IND; 4 Medicine, Lala Lajpat Rai Memorial College, Ghaziabad, IND

**Keywords:** brachial plexus anatomic variation, brachial plexus block, median nerve variation in its formation and course, median nerve variation in its formation & course, quadruple roots, triple roots

## Abstract

The brachial plexus demonstrates a wide range of anatomical variations, many of which carry significant implications for clinical practice. Conventionally, the median nerve arises from two roots: a lateral root from the lateral cord and a medial root from the medial cord. During the routine dissection of a 72-year-old female cadaver, an unusual bilateral but asymmetric anomaly was observed. On the left side, the median nerve was formed by three roots, while on the right, it was formed by four roots. In both axillae, the variant roots united anterior to the third part of the axillary artery. No additional plexus abnormalities were present. The coexistence of triple and quadruple roots in the same individual is exceptionally rare. These variations may complicate axillary surgeries, reduce the reliability of brachial plexus blocks, and predispose to unexpected neurological deficits. Recognition of such anomalies is essential for surgeons, anesthesiologists, neurologists, and anatomists.

## Introduction

The brachial plexus, formed by the anterior rami of C5 to T1 spinal nerves, provides motor and sensory innervation to the upper limb through a complex organization of roots, trunks, divisions, cords, and terminal branches [1,2]. Among its terminal branches, the median nerve typically originates from two roots: the lateral root arising from the lateral cord and the medial root arising from the medial cord [1,3].

However, numerous variations in the formation of the median nerve have been documented, including additional roots, atypical patterns of communication with the musculocutaneous nerve, or delayed unions [4,5]. Such deviations are particularly relevant to clinicians, as they may complicate axillary lymph node dissections, vascular repairs, and fracture fixation procedures, while also affecting the efficacy of brachial plexus anesthesia [6-8].

Although triple-rooted and quadruple-rooted formations of the median nerve have individually been described, their coexistence in the same individual has rarely been reported [4,8]. This case report describes a unique bilateral but asymmetric variation, with the median nerve being formed by three roots on the left and four roots on the right, observed during routine cadaveric dissection.

## Case presentation

A 72-year-old female cadaver underwent routine dissection at Naraina Medical College and Research Center, Kanpur, India. Standard dissection protocols were followed using Gray’s Clinical Photographic Dissector as a reference guide.

**Figure 1:**
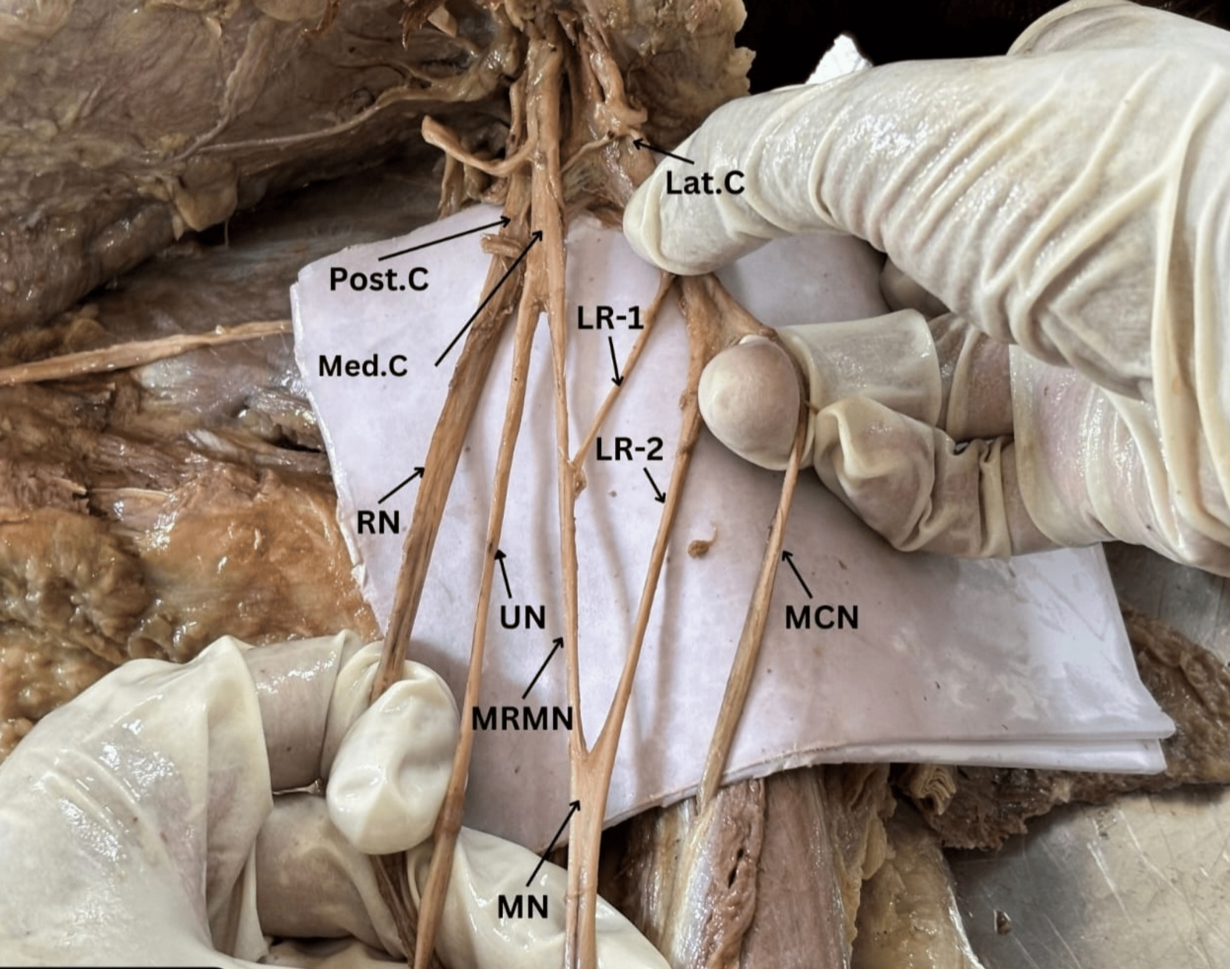
Two lateral roots originating from the lateral cord and one medial root originating from the medial cord form the three roots of the median nerve in this cadaveric section of the left axilla containing the brachial plexus Med. C: medial cord of the brachial plexus, Lat. C: lateral cord of the brachial plexus, Post. C: posterior cord of the brachial plexus, RN: radial nerve, MRMN: medial root of the median nerve, UN: ulnar nerve, MCN: musculocutaneous nerve, LR1, LR2: lateral root of the median nerve

Two lateral roots originating from the lateral cord and one medial root originating from the medial cord form the three roots of the median nerve in the cadaveric specimen of the left axilla with brachial plexus shown in Figure 1. Furthermore, the radial nerve (RN), medial root of the median nerve (MRMN), ulnar nerve (UN), musculocutaneous nerve (MCN), lateral root of the median nerve (LR1, LR2), medial cord of the brachial plexus (Med. C), lateral cord of the brachial plexus (Lat. C), and posterior cord of the brachial plexus (Post. C) are shown in Figure 1, highlighting the anatomical relationships and variations that can occur in this complex network of nerves.

**Figure 2:**
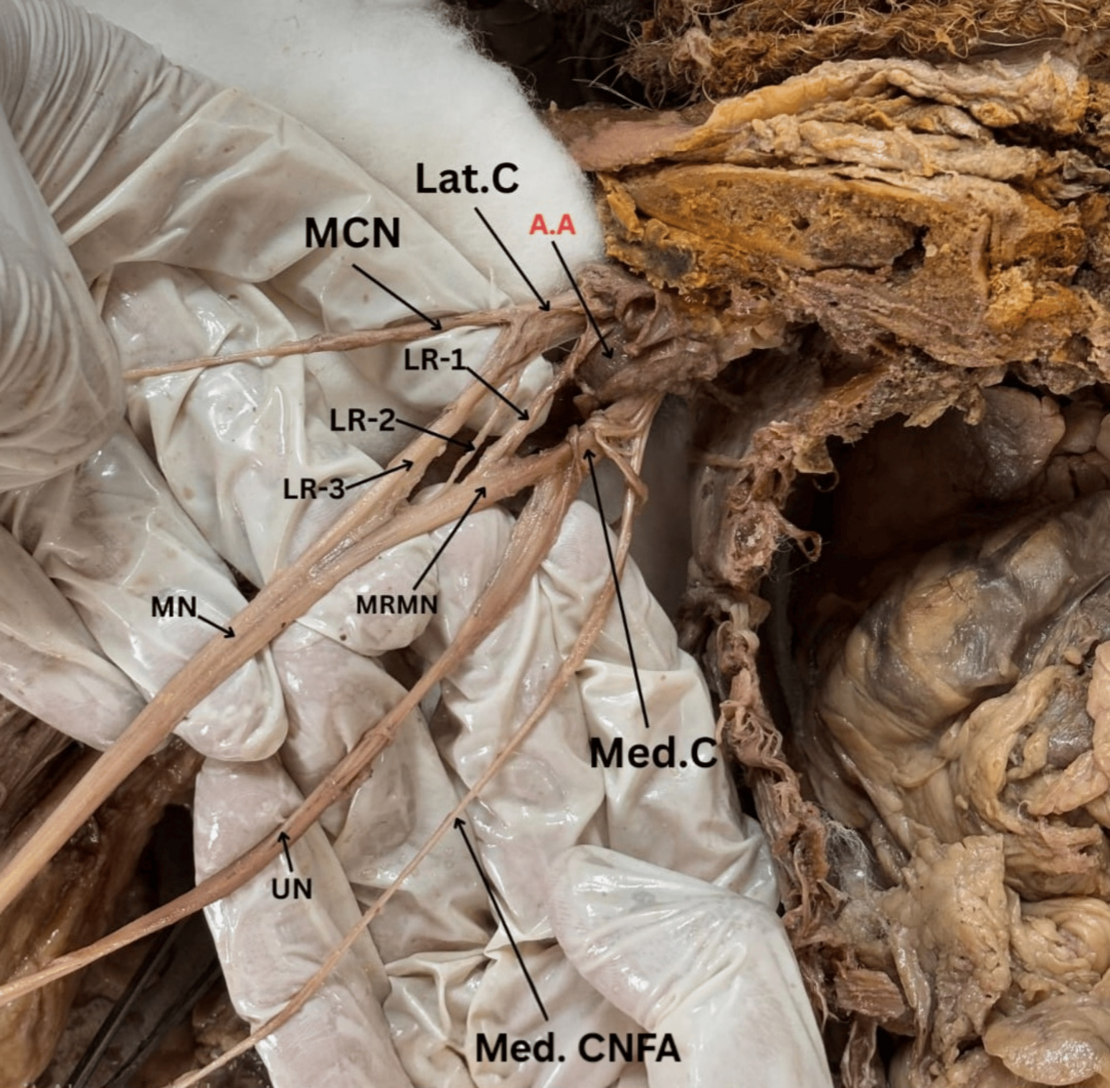
The median nerve shows four roots, three of which come from the lateral cord, while the fourth originates from the medial cord, as seen in this cadaveric section of the right axilla accompanying the brachial plexus Med. C: medial cord of the brachial plexus, Lat. C: lateral cord of the brachial plexus, Med CNFA: medial cutaneous nerve of the forearm, MRMN: medial root of the median nerve, UN: ulnar nerve, MCN: musculocutaneous nerve, MN: median nerve, LR1, LR2, LR3: lateral root of the median nerve, A.A: axillary artery

The median nerve in the right axilla was formed out of four roots, three of which came from the lateral cord along with one of the medial cords, as depicted in the cadaveric specimen of the right axilla with brachial plexus in Figure 2. The medial cutaneous nerve of the forearm (Med CNFA), MRMN, UN, Med. C, and Lat. C are also shown in Figure 2 along with the axillary artery (A.A.), lateral root of the medial nerve (LR1, LR2, LR3), MCN, and median nerve (MN). In both cases, the variant roots joined anterior to the third part of the axillary artery, ultimately forming the median nerve trunk. No additional anomalies of the brachial plexus were identified.

## Discussion

Variations in the formation of the median nerve are not uncommon, though they most often involve an additional lateral root [5,8]. Reports of triple-root formations are rare, and the presence of four roots is even less frequent [6]. The simultaneous occurrence of both patterns in the same individual represents an exceptional anomaly.

From a clinical standpoint, such variations have important implications. The success of regional anesthesia, particularly brachial plexus blocks, relies heavily on predictable nerve anatomy. The presence of accessory roots may result in incomplete anesthesia or unusual patterns of motor or sensory sparing [4]. Similarly, during axillary lymph node dissections or vascular repairs, atypical root configurations increase the risk of inadvertent nerve injury [7,9]. Orthopedic interventions around the shoulder and humerus, including fracture fixation, may also be complicated by altered neural pathways [5].

Neurologically, anomalous root contributions could lead to variations in axonal supply, which may predispose individuals to atypical patterns of weakness or sensory disturbance in muscles and skin regions innervated by the median nerve [6,8]. Embryologically, these anomalies can be attributed to altered signaling during limb bud development [10]. Molecular cues and transcription factors guide axonal growth into the upper limb; disturbances in these processes may lead to duplication or splitting of roots, thereby accounting for the observed variation [10,11].

This case underscores the need for clinicians and anatomists to remain vigilant regarding the diversity of peripheral nerve anatomy. Reports of bilateral but asymmetric anomalies, such as the coexistence of triple and quadruple roots, emphasize the unpredictable nature of brachial plexus development and highlight the importance of incorporating such knowledge into surgical training and practice [12].

## Conclusions

This case report documents a rare bilateral but asymmetric anomaly in the formation of the median nerve, with three roots on the left and four roots on the right. Awareness of such variations is essential for surgeons, anesthesiologists, and neurologists to minimize intraoperative complications, improve the success of regional anesthesia, and ensure accurate interpretation of neurological deficits. For anatomists and educators, such findings reinforce the importance of emphasizing anatomical variability during medical teaching.

## References

[1] Drake RL, Vogl W, Mitchell AWM, Gray H (2020). Gray's Anatomy for Students.

[2] Bala A, Sinha P, Tamang BK, Sarda RK (2014). Anatomical variation: median nerve formation - a case vignette. J Clin Diagn Res.

[3] Dalley AF, Agur AM (2023). Moore’s Clinically Oriented Anatomy.

[4] Patil S, Rathinam B, Kumar B, Chaware P, Wakode N, Wakode S, Gandhi K (2023). A cadaveric study to define the variant patterns of median nerve formation. Cureus.

[5] Akhtar MJ, Kumar S, Chandan CB, Kumar B, Sinha RR, Akhtar MK, Kumar A (2022). Variations in the formation of the median nerve and its clinical correlation. Maedica (Bucur).

[6] Bozhikova E, Novakov S, Petleshkova T, Harizanova Z, Uzunov N (2025). Variations in the terminal branches of the brachial plexus in the axillary fossa. Biomed Res Int.

[7] Ballesteros LE, Forero PL, Buitrago ER (2015). Communication between the musculocutaneous and median nerves in the arm: an anatomical study and clinical implications. Rev Bras Ortop.

[8] Soubeyrand M, Melhem R, Protais M, Artuso M, Crézé M (2020). Anatomy of the median nerve and its clinical applications. Hand Surg Rehabil.

[9] Satyanarayana N, Vishwakarma N, Kumar GP, Guha R, Datta AK, Sunitha P (2009). Variation in relation of cords of brachial plexus and their branches with axillary and brachial arteries--a case report. Nepal Med Coll J.

[10] Saddler TW (2006). Langman’s Medical Embryology.

[11] Encarnacion M, Nurmukhametov R, Barrientos RE (2022). Anatomical variations of the median nerve: a cadaveric study. Neurol Int.

[12] Nichols AM, Patel DB, Geske NL, McMillan W (2023). A case report on brachial plexus anomaly, embryological basis, and clinical implications. Cureus.

